# Laser Synthesis of Nanomaterials

**DOI:** 10.3390/nano12172903

**Published:** 2022-08-24

**Authors:** Mohamed Boutinguiza Larosi, Jesús del Val García, Antonio Riveiro Rodríguez

**Affiliations:** 1 Department of Applied Physics, University of Vigo, EEI, Lagoas-Marcosende, 36310 Vigo, Spain; 2 LaserON Laser Applications Research Group, Research Center in Technologies, University of Vigo, Energy and Industrial Processes, Rúa Maxwel, 36310 Vigo, Spain; 3 Defense University Center at the Spanish Naval Academy, University of Vigo, Plaza de España 2, Marín, 36920 Pontevedra, Spain; 4 Materials Engineering, Applied Mechanics and Construction Department, University of Vigo, EEI, Lagoas-Marcosende, 36310 Vigo, Spain

Nanomaterials, defined as materials with typical dimensions of less than 100 nm in at least one dimension, exhibit very special physicochemical properties that are highly dependent on their size and shape. These new properties, different from those of the corresponding bulk material, are behind the revolution in both nanomaterial fabrication techniques and the application of nanomaterials in many areas of science and technology. The possibility of tailoring the properties of materials by manipulating them at the nanoscale to obtain materials with highly specific surface area and improved mechanical, optical, magnetic, etc., properties has opened up and promoted new and promising lines of research. Assembling nanostructures by different methods, called nanofabrication, has led to enormous growth in the majority of industrial sectors.

There are many techniques and methods for producing nanostructures. These can be broadly classified into: “top down” methods, in which the starting material is reduced to the desired sizes following physical or chemical methods (such as electron or ion beam, milling, laser ablation, and reactive ion etching), and “bottom-up” methods, in which nanofabrication is carried out by assembling individual atoms or molecules to obtain the final nanostructure; this strategy includes chemical and physical vapor deposition (CVD and PVD), epitaxial growth, self-assembly, etc. [1,2].

Among the wide range of nanofabrication techniques, laser ablation is a nanomaterial synthesis technique with outstanding advantages, such as high production efficiency, low cost, good stability, and reliable processing quality, in addition to being environmentally friendly. This technique has been used to fabricate a wide range of nanomaterials and nanostructures with improved chemical, optical, magnetic, and electronic properties. Laser ablation technique has been used in a variety of experimental setups and configurations to synthesize different nanomaterials in a wide range of atmospheres and conditions. Laser-based techniques, such as laser ablation, laser vaporization, pulsed laser deposition (PLD), laser–chemical vapor deposition (LCVD), etc., are being used to fabricate nanoscale materials with a controlled size, shape, and specific properties [3]. Another laser-based nanomaterial processing technique with major impact in nanotechnology is laser synthesis and processing of colloids (LSPC), a scalable and versatile technique for the synthesis of ligand-free nanomaterials in controlled liquid environments. With this method, nanoparticles with high surface purity can be synthesized and alloys or series of doped nanomaterials can also be obtained. This technique can be classified into three approaches: laser ablation in liquids (LAL), where the laser beam is kept scanning a bulk target in liquid to produce colloidal nanoparticles.; laser fragmentation in liquids (LFL), in which microparticle suspensions or colloidal nanoparticles are irradiated with lasers to be fractured; and laser melting in liquids (LML), in which the laser beam is used to melt primary nanoparticles into bigger ones [4]. In this context, the present Special Issue has been organized to include works focused on obtaining nanostructured materials by laser-based synthesis methods. Combined properties of different starting materials can be also obtained by laser synthesis, as is reported by Alexey Rybaltovsky et al. [5]. Two approaches are used to synthesize bimetallic Au/Ag nanoparticles, using a laser to ablate targets of gold and silver in a medium of supercritical carbon dioxide. In the first configuration, Ag and Au targets are placed side-by-side, vertically, on the side wall of a high-pressure reactor, and the ablation of the target plates occurs alternately with a stationary “wide” horizontal beam with a laser pulse repetition rate of 50 Hz. With this configuration, Ag/Au alloy nanoparticles are obtained. Meanwhile, “core–shell” bimetallic Au/Ag nanoparticles with a gold core and a silver shell are synthesized by placing the targets horizontally at the bottom of a reactor, and the ablation of their parts is carried out by scanning from above with a vertical “narrow” laser beam with a pulse repetition rate of 60 kHz.

Roman Romanov et al. [6] conducted an investigation to produce MoSx~4/WSe2/C(B)/Al_2_O_3_ photocathodes using PLD, which can be employed for effective solar water splitting to produce hydrogen. The fabricated photocathode presents the following characteristics as regards hydrogen evolution reaction in 0.5 M H_2_SO_4_ acid solution during light irradiation with an intensity of 100 mW/cm^2^: the current density at 0 V (RHE) is ~3 mA/cm^2^; the onset potential reaches 400 mV (RHE).

The synthesis of air-stable Cu nanoparticles by irradiating solutions of copper acetylacetone in a mixture of methanol and isopropyl alcohol by femtosecond laser is reported by Ashish Nag et al. [7] following the method of bottom-up laser reduction in liquid. The obtained Cu NPs exhibit remarkable stability over 7 days on the basis of the lack of significant changes observed in the UV-vis absorbance and XRD features, and their photocatalytic performance was maintained.

Izumi Takayama et al. [8] reported the fabrication of hollow channels surrounded by gold nanoparticles in poly(ethylene glycol) diacrylate (PEGDA). The hollow channels and gold nanoparticles were formed in a single step of irradiating a femtosecond laser pulse in PEGDA hydrogels which contained gold ions. Taking into account that hydrogels and gold nanoparticles present high biocompatibility, this research could open the door to many applications in tissue engineering, microfluidics, and drug delivery.

The structure and chemical states of thin-film coatings obtained by pulsed laser co-deposition of Mo and C in a reactive gas (H_2_S) are investigated [9]. The performance of these coatings was analyzed for their prospective use as solid lubricating coatings for friction units operating in extreme conditions, showing that the nanophase composition in Mo–S–C–H_5.5 coatings has good antifriction properties and increased wear resistance, even at −100 °C.

An approach for producing porous plasmonic nanostructures using direct ns-laser ablation followed by Ar-ion beam etching is reported in Ref. [10]. The nanopores were found to form through the explosive evaporation/boiling of the nitrogen-rich metal film areas irradiated by an ns laser pulse. This scalable method for producing 3D plasmonic nanosponges represents a promising technique to control and improve their performances for various nonlinear optical and sensing applications.

Iron oxide nanoparticles are synthesized by laser ablation in water by María J. Rivera-Chaverra et al. [11]. Their characteristics, as a function of the laser energy and for the possible application in magnetic hyperthermia, were evaluated. It was shown that the temperature rise in iron oxide nanoparticles was not greatly influenced by the energy change in magnetic hyperthermia measurements. Experiments show that, for hyperthermia applications, low values of laser energy give better results, as these produce higher specific absorption rate (SAR) values.

Li-Hsiou Chen et al. [12] reported the synthesis of Graphene (Gr)/gold (Au) and graphene-oxide (GO)/Au nanocomposites (NCPs) by pulsed-laser-induced photolysis (PLIP) on hydrogen peroxide and chloroauric acid (HAuCl4) that coexisted with Gr or GO in an aqueous solution. Both kinds of nanoparticles exhibited photocatalytic degradation of methylene blue under solar light illumination with removal efficiencies over 92% and showed good stability and a large potential in the practical treatment of dye-contaminated wastewater through an ecofriendly fabrication process.

Kristin Charipar et al. [13] used pulsed laser fragmentation in liquid as a ligand-free alternative to traditional nanoparticle synthesis techniques for the fabrication of ZnO nanoparticles. They also demonstrated the possibility of producing hybrid ZnO nanoparticle/graphene phototransistors, exhibiting a responsivity of up to 4 × 10^4^ AW^−1^ with a maximum gain of 1.3 × 10^5^ and superior spectral selectivity below 400 nm, which make them ideal for solar-blind UV photodetectors.

The carbonaceous flake-like structures self-assemble during a laser-induced growth process is studied in Ref. [14], with particular emphasis to the dependence of the optical and geometrical properties of these hybrid carbon–metal flakes on the fabrication parameters. This study shows that the geometrical parameters of orthorhombic metal–carbon hybrid flakes can be tailored during the fabrication process by controlling various fabrication parameters, such as irradiation time and the application of an external field along the substrate.

Yanwei Huang et al. [15] reported the synthesis of Zn-doped calcium copper titanate (CCTO) by rapid laser sintering of sol–gel-derived precursors without the conventional long-time heat treatment. The used technique overcomes the shortcomings of long-time thermal energy supply by a furnace and presents references to synthesize the ceramic materials through a combination of soft-chemical methods.

Mónica Fernández-Arias et al. [16] reported the synthesis of Cu and Cu oxide nanoparticles by laser ablation in open air and in argon atmosphere using 532 and 1064 nm radiation generated by nanosecond and picosecond Nd:YVO_4_ lasers, respectively, to be directly deposited onto Ti substrates. The coatings were tested as an antibacterial agent, showing strong antibacterial activity of the obtained copper nanoparticles against *S. aureus*. The best inhibitory effects are provided by Cu nanoparticles obtained by laser ablation in argon with a laser radiation of 1064 nm in wavelength. These results confirm the influence of size, crystallographic structure, and oxidation state in the bactericidal effects of copper nanoparticles.

Two modes of PLD from a MoS2 target were reported by V. Fominski et al. [17] to obtain amorphous MoSx-based catalysts with different compositions and morphologies. The mode of off-axis PLD differs from the mode of on-axis PLD in terms of a higher deposition rate for catalytic MoSx~3 + δ films and a larger S concentration in the amorphous MoSx~3 + δ (δ ~0.8–1.1) phase.

Maurizio Muniz-Miranda et al. [18] reported the synthesis of magneto-plasmonic nanoparticles constituted of gold and iron oxide in an aqueous environment by laser ablation of iron and gold targets in two successive steps without the presence of surfactants, stabilizers, or any contaminants. The plasmonic properties of the obtained colloids, as well as their adsorption capability, were tested by surface-enhanced Raman scattering (SERS) spectroscopy using 2,2′-bipyridine as a probe molecule.

Laser-based synthesis techniques emerged as powerful and versatile technology for nanomaterials synthesis, proving their feasibility for the synthesis of nanoparticles and nanostructures from different starting materials (metals, oxides, semiconductors, etc.) in a wide range of liquid and gas environments. This Special Issue presents different works using different laser-based techniques and approaches to tailor the final nanomaterial in order to obtain specific properties to improve certain applications, such as SERS, photocatalysis, photodetectors, hyperthermia, or antibacterial agents.

However, despite the relatively simple experimental setup of laser-based synthesis of nanomaterials, there are many limitations and challenges to be overcome, such as the limited productivity, adequate nanomaterials adhesion onto the appropriate substrate, better understanding laser beam–target interactions and removal mechanisms to better control the final product, higher stability in the case of colloidal solutions, etc.

Given the increasing number of researches and the rapid evolution of the laser synthesis of nanomaterials field, it is expected that most of the mentioned disadvantages will be reduced or overcome in the near future by improving the aforementioned techniques and/or integrating them with other synthesis routes.
